# Strategies for expanding vaccination coverage in children in Brazil: systematic literature review

**DOI:** 10.1590/0034-7167-2023-0343

**Published:** 2024-12-16

**Authors:** Janaina Fonseca Almeida Souza, Thales Philipe Rodrigues da Silva, Camila Kümmel Duarte, Anna Luiza de Fatima Pinho Lins Gryschek, Elysângela Dittz Duarte, Fernanda Penido Matozinhos

**Affiliations:** I Universidade Federal de Minas Gerais. Belo Horizonte, Minas Gerais, Brazil; II Secretaria de Estado de Saúde de Minas Gerais. Belo Horizonte, Minas Gerais, Brazil; III Universidade Federal de São Paulo. São Paulo, São Paulo, Brazil; IV Universidade de São Paulo. São Paulo, São Paulo, Brazil

**Keywords:** Immunization, Child, Vaccination Coverage, Health Communication, Politics., Inmunización, Niño, Cobertura de Vacunación, Estrategias de Salud, Política.

## Abstract

**Objectives:**

to identify the strategies found in the literature for increasing vaccination coverage among children in Brazil. It is justified mainly by the current scenario of falling vaccination coverage.

**Methods:**

systematic literature review. The search was carried out in the Pubmed (MEDLINE), Embase and Scopus databases, following the PRISMA guidelines.

**Results:**

initially, 4,824 results were returned. In the end, 6 studies were included for narrative synthesis using the SWiM methodology. Of these, 50% dealt with studies related to the *Bolsa Família* Program (PBF). The others explored strategies for approaching parents directly, Rapid Vaccination Monitoring (MRV) and the Community Health Agents Program (PACS). The PBF did not guarantee compliance with the conditionality of keeping vaccinations up to date. The MRV and PACS are effective strategies, especially because they allow active search for absentees.

**Conclusions:**

we conclude that more publications are needed on strategies to increase vaccination coverage among children in Brazil.

## RESUMEN

**Objetivos:**

identificar las estrategias encontradas en la literatura para aumentar la cobertura de vacunación infantil en Brasil. Se justifica principalmente por el escenario actual de caída de la cobertura de vacunación.

**Métodos:**

revisión sistemática de la literatura. La búsqueda se realizó en las bases de datos Pubmed (MEDLINE), Embase y Scopus, siguiendo las directrices PRISMA.

**Resultados:**

inicialmente se obtuvieron 4.824 resultados. Finalmente, se incluyeron 6 estudios para la síntesis narrativa mediante la metodología SWiM. De ellos, el 50% se referían a estudios relacionados con el *Programa Bolsa Família* (PBF). Los demás exploraban estrategias de aproximación directa a los padres, el Seguimiento Rápido de la Vacunación (MRV) y el Programa de Agentes Comunitarios de Salud (PACS). El PBF no garantizó el cumplimiento de la condicionalidad de mantener las vacunas al día. El MRV y el PACS son estrategias eficaces, sobre todo porque permiten la búsqueda activa de ausentes.

**Conclusiones:**

se concluye que son necesarias más publicaciones sobre estrategias para aumentar la cobertura de vacunación infantil en Brasil.

## INTRODUCTION

**Figure 1:**
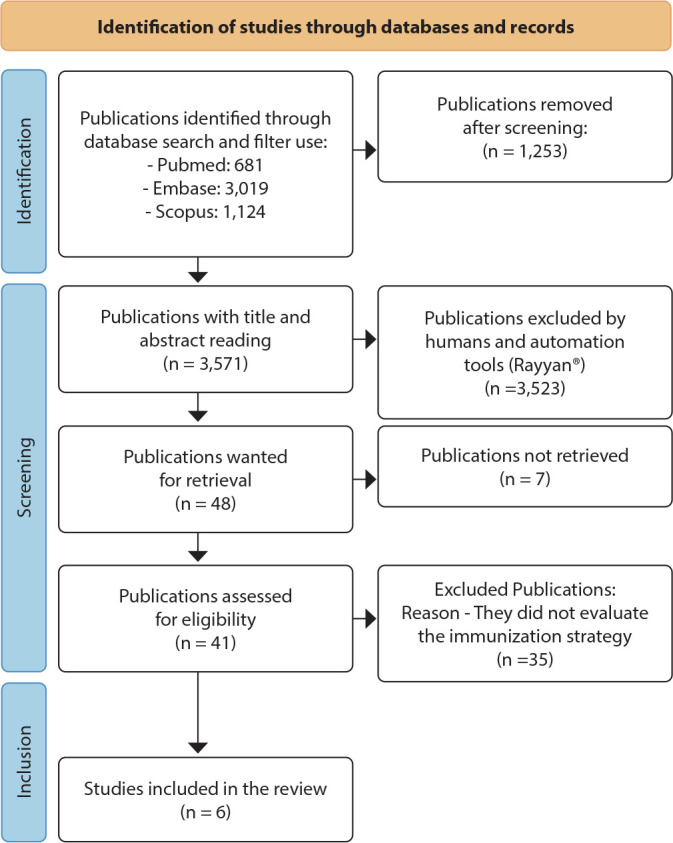
Article selection process

*Adapted from: Page MJ, McKenzie JE, Bossuyt PM, Boutron I, Hoffmann TC, Mulrow CD, et al. The PRISMA 2020 statement: an updated guideline for reporting systematic reviews. BMJ 2021;372:n71. doi: 10.1136/bmj.n7*

Vaccines are one of the greatest public health achievements in the 20th century, being a safe and cost-effective intervention for the control, eradication or elimination of vaccine-preventable diseases^(1,2)^.

In the Brazilian context, the National Immunization Program (PNI), coordinated by the Ministry of Health, in a shared manner with the state and municipal health departments, has consolidated itself as one of the most relevant public health interventions^(3)^. Created in 1973, the PNI has a history of achievements and challenges, and is characterized as an efficient public policy, increasingly impacting the morbidity and mortality profile of the Brazilian population, adapting to changes in the political, epidemiological and social fields^(3)^.

In recent decades, several vaccine-preventable diseases have been eliminated or had a considerable reduction in the number of cases and deaths, thanks to vaccination. Paradoxically, great challenges have arisen for the PNI, despite all the achievements. Many diseases have become unknown, causing some people to no longer have dimension and knowledge of the severity represented by them, with consequent risk of reintroduction or recrudescence of controlled or already eradicated diseases in the country^(4,5)^. A new phenomenon began to be seen in several places around the world: the reduction in the achievement of the recommended targets for vaccination coverage rates^(6)^. In Brazil, this phenomenon is observed especially from 2016^(5)^.

The low vaccination coverage rates with the MMR vaccine (protection against measles, mumps and rubella), for example, contributed to the accumulation of individuals susceptible to viruses and the return of measles to Brazil, especially in 2018^(7)^. The country lost the title of area free of the autochthonous virus circulation it had received in 2016^(5)^.

According to an ecological study^(8)^ involving the analysis of vaccination coverage in children up to 12 months of age in the period from 2013 to 2020, it is possible to notice the significant reduction of this indicator over the years, intensifying even more with the COVID-19 pandemic. In this context, one can see the vaccination coverage already had values below the targets established by the Ministry of Health and, each year, the fall is accentuated^(8)^.

The reduction in vaccination coverage rates in recent years cannot be attributed to a single cause^(9)^. Several factors can affect the fall in vaccination coverage in children. Contextual, historical, sociocultural, environmental, health system, economic, political, and even individual factors can be highlighted. Understanding these associations is a complex process^(7,10-12)^, but it is essential to recognize the multiple factors to develop strategies to increase vaccination coverage.

Given the complex scenario of falling vaccination coverage that is installed in Brazil and worldwide (and which was even more impacted by the COVID-19 pandemic), with reasons described for the failure of the current measures, and with proof of the difficulties faced by the PNI, there is a growing concern with the creation of strategies aimed at increasing vaccination coverage among children, especially with improved access to health services, regardless of the type of vaccine applied and the social characteristics of younger children^(13)^.

Interventions to improve vaccination outcomes are commonly grouped into those aimed at providing or delivering health services (e.g., improving human resource training, logistics, cold chain maintenance and vaccine storage, effective financing, monitoring, and supportive supervision) and those that stimulate demand for vaccines (monetary or food incentives, knowledge transfer, or communication campaigns). Systematic reviews at international level that showed these studies were found^(14,15)^, but with limitations in quality and design^(16)^. The Secretariat of State for Health of the State of Goiás, Brazil, recently released an overview of systematic reviews covering strategies to increase vaccination coverage. Among the 18 systematic reviews published between 2018 and 2021, no reviews on this theme were identified in Brazil, which corroborates the need for studies that implemented strategies at the national level^(17)^.

This work can, therefore, subsidize the health administrators and professionals involved with vaccination in the construction of the planning and implementation of their actions, since the Brazilian PNI is considered one of the most complete in the world^(7)^.

## OBJECTIVES

To identify the strategies found in the literature for increasing vaccination coverage among children in Brazil, without restricting the period. It is justified mainly by the current scenario of falling vaccination coverage.

## METHODS

This is a systematic literature review carried out in accordance with the recommendations of the *Cochrane Handbook for Systematic Intervention Reviews* ^(18)^ and reported based on the steps recommended by PRISMA^(19)^. The study protocol was registered on the OSF^®^ Platform (Open Science Framework), under the identifier 10.17605/OSF.IO/XHM6P: DOI^(20)^.

The research question of this review was: “What are the strategies used to increase vaccination rates in Brazil?”. To adapt the research question to the construction of the search strategy, the PCC structure (Problem - Concept - Context) was used^(21)^ (Table S1 - Research Data). For the acronym P (Population), children were taken into account. For the acronym C (Concept), the descriptors health planning, action plan and municipal management were used. For the acronym C (Context), the descriptors vaccination, immunization, vaccination coverage, Immunization Program and Brazil were used.

The option to consider only strategies implemented in Brazil was due to the fact that the Brazilian PNI is considered one of the most complete in the world, with international recognition, high vaccination coverage for several diseases of importance to public health, as well as the implementation of strategies and logistics to achieve immunization for the entire Brazilian population, which is inserted in an extensive continental territory^(7)^.

### Search strategy

The search strategy was performed with the PCC terms adapted to the different PubMed (MEDLINE), Embase and Scopus databases (Table S2 - Research Data). The terms of the PCC methodology, when available as index terms (MESH and Entree), were used concomitantly with the terms text^(22,23)^. The searches were conducted without restrictions on year and language. The search for data was carried out in December 2022 and updated in April 2023 through a new search. The synonymous search terms included were separated by Boolean operators “OR” and each group of PCC synonymous terms grouped by “AND”.

The search keys used in each database are described below:

Pubmed: (((“Child”[MeSH Terms] OR “Child”[MeSH Terms] OR “Child”[TW] OR “children”[TW] OR “child s”[TW] OR “children s”[TW] OR “childrens”[TW] OR “childs”[TW] OR “Child”[MeSH Terms] OR “Child”[TW] OR “children”[TW] OR “child s”[TW] OR “children s”[TW] OR “childrens”[TW] OR “childs”[TW] OR “Infant”[MeSH Terms] OR “Infant”[MeSH Terms] OR “Infant”[TW] OR “infants”[TW] OR “infant s”[TW] OR “Infant”[MeSH Terms] OR “Infant”[TW] OR “infants”[TW] OR “infant s”[TW] OR “nurseling”[TW] OR “nursling”[TW] OR “nurslings”[TW])) AND (((“Health Planning” OR “Health Management” OR “Municipal Management” OR “Action Plan”) AND (“Vaccination”[Mesh] OR (Vaccination) OR “Immunization”[Mesh] OR (Immunization) OR (Vaccination Coverage) OR (Immunization) OR (Active Immunization) OR (Immunization Coverage) OR “Mass Vaccination”[Mesh] OR (Mass Vaccination) OR “Vaccination Coverage”[Mesh] OR (Vaccination Coverage))) OR ((Immunization strategies) OR (Immunization programs) OR “Immunization Programs”[Mesh]))) AND (“Brazil”[Mesh] OR (Brazil) OR (Brasil))

Embase: (‘child’/syn OR ‘infant’/syn) AND (‘vaccination’/syn OR ‘immunization’/syn OR ‘mass immunization’/syn OR ‘preventive health service’/syn) AND (‘Brazil’/syn)

Scopus: ( ALL ( children OR child OR infant ) ) AND ( ALL ( “Health Planning” OR “Health Management” OR “Municipal Management” OR “Action Plan” ) ) AND ( ALL ( “Vaccination” OR “Immunization” OR “Vaccination Coverage” OR “Active Immunization” OR “Immunization Coverage” OR “Mass Vaccination” ) ) AND (ALL (Brazil))

### Eligibility Criteria

Observational studies that contained strategies to increase vaccination coverage in children and that were carried out in Brazil, without period restriction, were included. Exclusion criteria were (1) Systematic reviews and meta-analyses; (2) Clinical trials; (3) Experimental studies; (4) Case-control studies; (5) Letters to the Editor; (6) Studies that did not present intervention strategies in increasing vaccine coverage.

### Selection and Extraction of Studies

Two independent reviewers (JFAS and TPRS) examined the titles and abstracts of the articles identified through the Rayyan^®^ platform. Studies that met the inclusion criteria were submitted to full-text reading. Discrepancies were resolved by consensus. In the persistence of doubt, a third (CKD) and fourth reviewer (FPM) were called. Those that met the eligibility criteria were included in the review. The same two reviewers performed data extraction using the SWiM methodology.

The SWiM guideline consists of a nine-item checklist to promote transparent reporting: (1) Grouping of studies for synthesis; (2) Description of the standardized metric and the transformation methods used; (3) Description of synthesis methods; (4) Criteria used to prioritize results for summary and synthesis; (5) Investigation of heterogeneity in reported effects; (6) Certainty of evidence; (7) Methods of data presentation; (8) Results reports and (9) Synthesis limitations^(24)^.

### Methodological quality

All included studies were independently evaluated by the authors (JFAS and TPRS) for methodological quality and risk of bias, using the Newcastle-Ottawa scale, adapted for observational studies^(21,23)^. The studies were evaluated using the following domains: (i) sample size and representativeness (0-4 points); (ii) comparability between participants (0-2 points) and (iii) description of the immunization strategy (0-3 points). A study was considered low quality if it received less than seven stars and high quality if it received seven stars or more (Chart 3).

### Data analysis

Data were grouped by narrative synthesis and the results were formatted in tables descriptively, using the SWiM methodology.

## RESULTS

The various immunobiologicals in children from 0 to 6 years of age were analyzed. Studies considered the following locations: Brazil^(25)^; the states of Ceará^(26-28)^, Bahia^(26)^, Goiás^(26)^, Pará ^(26)^, Pernambuco^(26)^, São Paulo^(26,29)^; and the municipalities of São Luís (MA) and Ribeirão Preto (SP)^(30)^ (Chart 1).

**Chart 1:** Characteristics of authorship, location, sample size, study population, age and immunobiologicals analyzed

Author, Year	Location	Sample size	Study Population	Age	Immunobiologicals analyzed
Andrade *et al*., 2012^(25)^	Brazil	7,550 children	(1) families classified in the poverty line, with children under 15 years of age or pregnant women; (2) families classified in the extreme poverty line with or without children.	Children from 0 to 6 years	Immunobiologicals that made up the Basic Vaccination Calendar of Children in the year of the study, up to 6 years of age.
Barcelos *et al.,* 2021^(26)^	30 municipalities of the states of Bahia, Ceará, Goiás, Pará, Pernambuco and São Paulo	3,242 children (T0) and 3,008 children (T1)	Children and pregnant women eligible for the Happy Child Program.	T0 - less than 12 months of life; T1 - median of 12 months	Immunobiologicals that made up the Basic Vaccination Calendar of Children in the year of the study, up to 6 years of age.
Costa *et al.,* 2020^(29)^	Municipality of São Paulo	151 children before the intervention and 145 after the intervention.	Members of the Center for Early Childhood Education of São Paulo (CEI)	Children from 0 to 4 years of age.	Immunobiologicals that made up the Basic Vaccination Calendar of Children in the year of the study, up to 4 years of age.
Moura *et al*., 2018^(27)^	State of Ceará	52,216 vaccination notebooks	Resident population in the State of Ceará	Children from 6 months to under 5 years of age	Double viral, triple viral and tetraviral vaccines.
Silva *et al*., 2020^(30)^	São Luís (MA) and Ribeirão Preto (SP)	532 children in Ribeirão Preto and 1,229 in São Luís	Beneficiaries: *Bolsa Família* Program users	Children from 13 to 35 months	Immunobiologicals that made up the Basic Vaccination Calendar of Children in the year of study, until the first year of life.
Cufino Svitone et al., 2000^(28)^	Ceará	-	Population of the state of Ceará covered by the Family Health Teams	-	-

Of the 6 articles included, 50% addressed studies related to the *Bolsa Família* Program (PBF) and evaluation of vaccination coverage^(25,26,30)^. The others explored strategies of direct approach to parents^(29)^, Rapid Vaccination Monitoring (MRV)^(27)^ and Community Health Agents Program (PACS)^(28)^ (Chart 2).

**Chart 2:** Characterization of strategies for increasing vaccination coverage

Author, Year	Strategy	Actor (who performed it)	Strategy description	Strategy level	Main results
Andrade *et al*., 2012^(25)^	Conditional Income Transfer Program (CCT)/*Bolsa Família* Program (PBF).	Federal Government	Establishment of conditionalities in income transfer programs so that families receive payment only if they meet certain requirements, such as keeping the children’s vaccination card up to date.	Macropolitics	PBF did not influence the vaccination status of children in 2005. Effects were found only in the Southeast/South region. On average, the effect of the program represents a 7% increase in immunization, with 10% being significant in this region.
Barcelos *et al.,* 2021^(26)^	Conditional Income Transfer Program (CCT)/Family Grant Program (PBF) /Happy Child Program (PCF)	Ministry of Civil Rights of Brazil	The PCF is directed to children under 3 years of age children of families benefited by the PBF. To receive the cash payment, registered parents must take all children under 7 years to the health unit for complete immunization.	Macropolitics	Low percentage of children with adequate vaccination, both in the first and second year of life. Belonging to the richest quintile - within a predominantly poor sample, was a factor associated with higher proportions of adequate vaccination at T0. At T1, vaccination coverage in the PCF group was higher than in the control group.
Costa *et al*., 2020^(29)^	Direct Approach to Parents	Members of the Center for Early Childhood Education of São Paulo (CEI)	Sending a reminder to families through the child’s agenda about the vaccination situation and the care that promotes their vaccination.	Micropolitics	There was a difference in the prevalence of vaccine completeness of delayed children after the intervention (increase of 11.6% - from 81.5% to 93.1%). Sending a reminder to parents: increasing the completion of children’s vaccinations.
Moura *et al*., 2018^(27)^	Rapid Vaccination Monitoring (MRV)	State Department of Health and municipalities of Ceará	MRV is characterized by seeking vaccination coverage in home visits to verify proof of vaccination.	Macropolitics	Identified 1.6% of unvaccinated children against measles. After MRV only four municipalities (Baturité, Itapipoca, Brejo Santo and Crato) did not reach vaccination coverage for the first dose, and two Cres (Itapipoca and Russas) did not reach vaccination coverage for the second dose.
Silva *et al*., 2020^(30)^	Conditional Income Transfer Program (CCT)/*Bolsa Família* Program (PBF).	Federal Government	Establishment of conditionalities in income transfer programs so that families receive payment only if they meet certain requirements, such as keeping the children’s vaccination card up to date.	Macropolitics	Being a PBF beneficiary had no effect on childhood vaccination in children belonging to low-income families, both in São Luís and Ribeirão Preto.
Cufino Svitone *et al*., 2000^(28)^	Community Health Agents Program - PACS	Government of the State of Ceará	Performance of Community Health Agents (Cha) in carrying out home visits and actions to prevent diseases and promote health in the community.	Macropolitics	Rapid decline in infant mortality, increase in immunization, identification of bottlenecks that limit the use of other medical resources, and timely interventions in times of CRIE. Rapid identification of areas with low vaccination coverage and problem solving.

**Chart 3:** Newcastle-Ottawa scale for analysis of study quality

Author, Year	Selection (maximum four stars)	Comparability (maximum two stars)	Result Rating (maximum three stars)	Quality
Andrade *et al*., 2012^(25)^	^****^	^**^	^***^	High
Barcelos *et al*., 2021^(26)^	^****^	^**^	^***^	High
Costa *et al*., 2020^(29)^	^**^	^*^	^**^	Low
Moura *et al.,* 2018^(27)^	^****^	^*^	^***^	High
Silva *et al*., 2020^(30)^	^****^	^**^	^***^	High
Cufino Svitone *et al*., 2000^(28)^	^**^	^*^	^***^	Low

The PBF, MRV and PACS represent strategies that are considered macro-political, that is, there is an institutional guideline with standardized instruments and tools for execution throughout the territory of the country. The strategy related to the direct approach to parents represented a micro-policy, that is, decisions were made by individuals, health agents from their territory, based on their local contexts and realities^(31)^.

Among the macro-policy strategies, the PBF stands out, which was addressed in three studies^(25,26,30)^. All of them evaluated the conditionalities of the PBF with the vaccination completeness of the participating children (Chart 2). In all three studies, the results were similar in that the PBF had no influence on improving vaccination coverage, even though it was one of the program’s conditionalities. In the study by Barcelos et al^(26)^, in addition to analyzing the percentage of children with adequate vaccination, a socioeconomic stratification was also carried out which showed that belonging to the richest quintile - in a predominantly poor sample - was a factor associated with higher proportions of adequate vaccination. What can be said, based on the three studies, is that it is important to improve both the monitoring of the program’s conditionalities and the monitoring of vaccination status, since the percentages of incomplete vaccination in children whose families receive the PBF were high.

In the study by Moura *et al*.^(27)^, MRV was defined as an activity to supervise vaccination actions recommended by the Pan American Health Organization (PAHO) since the 1980s, adopted in several countries in the Americas. The MRV also allowed us to know the reasons for non-vaccination, some of them being the responsibility of the children’s caregivers (refusal, loss of the vaccination booklet, lack of time) and others due to the responsibility of the management (difficulty of access to vaccination sites, lack of scheduling of the vaccine in the Children’s Health Booklet, lack of guarantee of the immunobiological stock, lack of flexibility in the opening hours of health units)^(27)^ (Chart 2).

The study by Svitone *et al*.^(28)^ reported the precursor strategy in the State of Ceará that gave rise to the Community Health Agents Program (PACS). As a result, PACS was widely used and enabled significant improvement in maternal and child health indicators between 1987 and 1994. Population monitoring allowed the rapid identification of areas with low vaccination coverage and coping with the problem. Among the keys to the success of the Program are the careful selection of agents and the quality of their supervision and training, carried out by nurses. It has become a model for eight neighboring Brazilian states^(28)^ (Chart 2).

The micro-policy strategy was reported by Costa *et al*.^(29)^, and the objective was to evaluate the completeness and vaccine delay of children from an early childhood education center (located in a region of high social vulnerability), before and after an educational intervention with families. The intervention consisted of sending a reminder to families through the child’s agenda about their vaccination status. If the completeness of the vaccination situation was verified, the family was congratulated for the care of the child and informed about the next vaccines to be taken. If there was a delay in the vaccination situation, the family was informed about the fact and instructed to seek a health service for regularization^(29)^ (Chart 2).

Regarding the assessment of the methodological quality of the studies, 4 studies^(25-27,30)^ were rated ≥ 7 out of 10 stars, obtaining a high quality rating. The studies by Costa *et al*.^(29)^ and Cufino Svitone *et al*.^(28)^ had low methodological quality, mainly in relation to the selection criteria (representativeness) and comparability (in this case, for any additional factor).

## DISCUSSION

The limited number of studies published in Brazil on strategies to increase vaccination coverage in children was evidenced through this systematic review. Most of the studies found dealt with a specific strategy, which is the *Bolsa Família* Program (PBF)^(25,26,30)^.

The PBF began in 2003, being an income transfer program created by the Brazilian Federal Government, with the objective of promoting the immediate reduction of poverty through direct income transfers^(31)^. It has an innovative character due to the possibility of breaking the intergenerational cycle of poverty historically experienced by the Brazilian population, especially the black and brown population. For this purpose, conditionalities were defined that reinforce the exercise of social rights in the areas of health and education, allowing, to a large extent, the fight against poverty in the future. Among the conditionalities is compliance with the National Child Vaccination Calendar^(32)^.

The main results of the three studies indicated that PBF did not contribute to the improvement of the children’s vaccination situation, although compliance with the Vaccination Calendar defined by the Ministry of Health is one of the conditionalities for receiving the benefit^(25,26,30)^.

The defense of the requirement of conditionalities for the PBF has been supported by the argument that it provides access to health, education and social assistance policies to those families benefiting from these programs. However, what is observed is that public policies are insufficient for the population, that is, it is not because these beneficiaries have to comply with certain actions that the policies will be fully ready to receive this population, as it should be^(33)^. It has been discussed that the conditions defined for public policies facilitate the access of the wealthier strata of society to basic health and education services, however, it is questioned about the ability of these services to adequately absorb (and supervise) the increase in demand resulting from compliance with the conditionalities^(34)^.

The results found on compliance with PBF conditionalities in Brazil differ greatly from those observed in Mexico, where they are systematically verified. For example, the Mexican Program requires children aged between 24 and 60 months to attend nutritional monitoring clinics every four months, in addition to involvement in preventive health and nutrition activities^(35,36)^. The authors reinforced the need to investigate the reasons for non-compliance with the Program’s conditionalities and the inspection costs associated with such public policy strategies^(25)^.

In the study by De Sousa Silva *et al*. ^(30)^, both in São Luís, Brazil, and in Ribeirão Preto, Brazil, among children belonging to low-income families, being a beneficiary of the PBF also had no effect on the childhood vaccination scheme. This study also cited the Mexican Program as a model that has demonstrated improved health outcomes, growth and child development.

Barcelos *et al*.^(26)^ considered some hypotheses for the difference found in the prevalence of vaccination coverage of their study: part of the decrease in coverage would reflect the lack of vaccines in the period (especially the pentavalent one); there are local characteristics in the implementation of the Immunization Program between states and municipalities (offer of vaccines, access to health services and regularity of registration in the Children’s Health Booklet), as well as in the adherence of families to the vaccination of their children. Considering vaccination is one of the conditionalities of PBF, differences can also be attributed to local characteristics of Program implementation. The findings of the study suggest the need for a detailed assessment of families’ adherence to conditionality to keep their children’s vaccination schedule updated^(26)^.

A study that researched the emergence of vaccine hesitancy among upper-class Brazilians concluded that, in recent years, the fastest decline in vaccination coverage is happening among children from wealthy families, probably related to vaccine hesitancy (i.e., a “delay in accepting or refusing vaccination despite the availability of vaccination services”)^(37)^. The growth of social media and the spread of fake news, combined with the current health services financing crisis, represents a major challenge for the increase in vaccination coverage^(38)^.

Despite being among the policy interventions with the greatest estimated impact on the drop in child mortality in the country, the PBF and the Family Health Strategy (ESF) are directly affected by fiscal austerity, which will substantially reduce spending on social assistance programs as a percentage of the country’s Gross Domestic Product (PIB)^(39)^. Constitutional Amendment 95 (EC 95) is the most impactful austerity measure, which is not limited to the economic crisis and will still last for 20 years, causing an important impact on the already fragile state of social welfare in Brazil^(40)^. The reduction in investment in health and social assistance programs (such as PBF) aggravated social inequality in the country, increased income concentration and deteriorated health indicators, including the vaccination coverage index^(40,41)^.

In the study by Moura *et al*.^(27)^, Rapid Vaccination Monitoring (MRV) was defined as an activity to supervise vaccination actions recommended by the Pan American Health Organization (PAHO) since the 1980s, adopted in several countries in the Americas. It consists of seeking vaccination coverage in home visits to verify proof of vaccination. The number of households visited is based on the size of the target population and the number of vaccine rooms in each municipality. The data were analyzed through a MRV implemented after an indiscriminate vaccination campaign against measles in the state of Ceará, in November 2014, obtaining satisfactory results.

Another descriptive study, also using data from the MRC after vaccination campaigns to eliminate measles and rubella in 2008, 2011 and 2012 in Brazil (children, adolescents and adults), reiterated the importance of maintaining this strategy for evaluating vaccination coverage, especially after intensification of vaccination actions. The MRC is able to rescue unvaccinated and direct interventions, especially at the local level. Provides, in a short time, the vaccination situation of the target population interviewed, and at a low cost, when compared to the costs of coverage surveys and the vaccination census^(3)^.

The active search strategy for absentees is cited as one of the most relevant to generate a positive impact on the increase in vaccination coverage. It is an attribution of the professionals of the family health team and has great relevance in the monitoring of users in risk situations and in the monitoring of the population in relation to the Vaccine Calendar^(42)^.

The study by Svitone *et al*.^(28)^ provides an account of the strategies used to implement the Community Health Agents Program (PACS) in the State of Ceará, highlighting the improvement of vaccination coverage after the use of this strategy. Throughout the SUS construction process, it is undeniable the importance of the implementation of PACS in building the link between the community and health services^(43)^. The work of community health agents involves cultural competence, community-guided thinking and the construction of a bond, in a daily relationship with the families in its area, sharing technical and popular knowledge^(44,45)^.

A study^(46)^ measured the knowledge of Community Health Agent (ACS) about the childhood vaccination schedule of children up to five years old. A quantitative, descriptive, before-and-after study was conducted with a sample of 25 CHWs from two Basic Health Units of Ananindeua-Pará. Before the training intervention, 76% of community health agents stated that they check the child’s vaccination card and give guidance to mothers; 96% think that checking the child’s vaccination card is part of their job and all community health agents (100%) stated that some mother or father has already asked the agent about their child’s vaccination card. These data reinforce the importance of the role community health agents in the active search and correct guidance on childhood vaccination, therefore, there is a need for investment in qualification and permanent education of these professionals^(46)^.

The study by Costa *et al*.^(29)^ differed from the other studies in this systematic review by addressing a micro-policy strategy, carried out at the local level. The sending of reminders and educational leaflets to families allowed the increase in the vaccination completeness of the children’s calendar, corroborating previous studies^(15,28,47,48)^ that implemented interventions with the objective of sharing knowledge about the importance of vaccination and helping parents remember the dates to vaccinate their children. The findings suggest that a simple and low-cost intervention can be implemented by nurses who invest in intersectoral actions, recommended by the Health at School Program^(28)^. Nursing practice in Primary Health Care involves monitoring the vaccination situation in early childhood education schools. This is an important strategy for the prevention of vaccine-preventable diseases and promotion of child health. A systematic review on vaccination programs in schools in high-income countries showed the role of nursing in monitoring the vaccination situation, as well as in the application of immunobiologicals and communication with parents^(49)^.

### Study limitations

The limitations of this study consist of having different sample sizes and the fact that they were carried out in different locations (which makes it difficult to compare the studies), but in the same country. Despite these limitations, this study has several strengths, such as the use of a rigorous methodology and the presentation of socio-economically similar populations. Another limitation concerns the fact that other databases were not used; however, the most recognized databases in the health area were used (Pubmed, Embase and Scopus). Again, the choice to only consider strategies implemented in Brazil was due to the fact that the Brazilian PNI is considered to be one of the most complete in the world, with international recognition.

### Contributions to the Field

This systematic review could help nurses involved in immunization to implement strategies to increase vaccination coverage in children, given the worrying scenario of the return of vaccine-preventable diseases and the scarcity of studies of this type in the area.

## CONCLUSIONS

This study corroborated the need to publish micro and macro-political strategies to increase vaccination coverage in children, especially because it is something inherent in federal, state and municipal services and requires greater visibility in academia.

The PBF is an important strategy, however, it requires greater supervision and monitoring of its conditionalities, especially compliance with the Child Vaccine Calendar. The fiscal austerity measures implemented in recent years have directly impacted the good indicators of this Program.

Rapid Vaccination or Coverage Monitoring (MRV/MRC) needs to be implemented in all vaccine intensification actions, as a strategy to verify the vaccination coverage of the territory, rescue the unvaccinated, and understand the reasons for refusal. One of the essential points of this strategy is the active search for absentees, which also needs to be part of the routine of Primary Health Care services.

The performance of community health agents (ACS) is undoubtedly an essential strategy for carrying out active searches for the unvaccinated and orientation of the population, since they represent the closest link between the health service and the community.

In addition to the macro-policy strategies reported, micro-policy strategies implemented according to the reality of each territory are also important, such as the integrated actions of health services and the school community.

The State, as a promoter of public health, must provide macro-policy strategies to increase vaccination coverage, ensuring constant monitoring and evaluation of its effectiveness and effectiveness. Health professionals involved with immunization at the local level, especially nurses, also have the duty to promote strategies based on their knowledge of the territory, implementing micro-policies that may be differential for the change of the local scenario.

## Data Availability

*https://doi.org/10.48331/scielodata.YRNMB1*
