# Supplementary material for: Strategies for expanding vaccination coverage in children in Brazil: systematic literature review
Source: Rev Bras Enferm. 2024 Dec 16;77(6):e20230343. doi: 10.1590/0034-7167-2023-0343 (PMC11654546; doi:10.1590/0034-7167-2023-0343)
Supplement: Translation

# Translation: Estratégias para ampliação das coberturas vacinais em crianças no Brasil: revisão sistemática de literatura

**DOI:** 10.1590/0034-7167-2023-0343pt

**Authors:** Janaina Fonseca Almeida Souza, Thales Philipe Rodrigues da Silva, Camila Kümmel Duarte, Anna Luiza de Fatima Pinho Lins Gryschek, Elysângela Dittz Duarte, Fernanda Penido Matozinhos

**Affiliations:** I Universidade Federal de Minas Gerais. Belo Horizonte, Minas Gerais, Brasil; II Secretaria de Estado de Saúde de Minas Gerais. Belo Horizonte, Minas Gerais, Brasil; III Universidade Federal de São Paulo. São Paulo, São Paulo, Brasil; IV Universidade de São Paulo. São Paulo, São Paulo, Brasil

**Keywords:** Imunização, Criança, Cobertura Vacinal, Estratégias de Saúde, Política.

## Abstract

**Objetivos:**

identificar quais foram as estratégias presentes na literatura para ampliação da cobertura vacinal em crianças no Brasil. Se justifica principalmente pelo cenário atual de queda de coberturas vacinais.

**Métodos:**

revisão sistemática de literatura. A busca foi realizada nas bases de dados Pubmed (MEDLINE), Embase e Scopus, seguindo as diretrizes PRISMA.

**Resultados:**

inicialmente, retornaram 4.824 resultados. Ao final, foram incluídos 6 estudos para síntese narrativa por meio da metodologia SWiM. Destes, 50% abordaram estudos relacionados ao Programa Bolsa Família (PBF). Os demais exploraram estratégias de abordagem direta aos pais, Monitoramento Rápido de Vacinação (MRV) e Programa de Agentes Comunitários de Saúde (PACS). O PBF não garantiu o cumprimento da condicionalidade de manter a vacinação em dia. O MRV e PACS são estratégias eficazes, especialmente porque permitem a busca ativa de faltosos.

**Conclusões:**

conclui-se que são necessárias mais publicações sobre estratégias para aumentar a cobertura vacinal em crianças no Brasil.

## INTRODUÇÃO

As vacinas referem-se a uma das maiores conquistas da saúde pública no século XX, sendo uma intervenção segura e de excelente custo-efetividade para controle, erradicação ou eliminação de doenças imunopreveníveis^(1,2)^.

No contexto brasileiro, o Programa Nacional de Imunizações (PNI), coordenado pelo Ministério da Saúde, de forma compartilhada com as secretarias estaduais e municipais de saúde, consolidou-se como uma das mais relevantes intervenções em saúde pública^(3)^. Criado em 1973, o PNI tem uma história de conquistas e desafios, e caracteriza-se como uma política pública eficiente, impactando cada vez mais o perfil de morbimortalidade da população brasileira, adequando-se às mudanças ocorridas nos campos político, epidemiológico e social^(3)^.

Nas últimas décadas, várias doenças imunopreveníveis foram eliminadas ou tiveram redução considerável no número de casos e óbitos, graças à vacinação. Paradoxalmente, grandes desafios surgiram para o PNI, a despeito de todas as conquistas. Muitas doenças tornaram-se desconhecidas, fazendo com que algumas pessoas não tenham mais dimensão e conhecimento da gravidade representada por elas, com consequente risco de reintrodução ou recrudescimento de doenças controladas ou já erradicadas no país^(4,5)^. Um novo fenômeno começou a ser visto em diversos locais do mundo: a redução no alcance das metas preconizadas para os índices de coberturas vacinais^(6)^. No Brasil, este fenômeno é observado especialmente a partir do ano de 2016^(5)^.

Os baixos índices de cobertura vacinal com a vacina tríplice viral (proteção para sarampo, caxumba e rubéola), por exemplo, contribuíram para o acúmulo de indivíduos suscetíveis aos vírus e o retorno do sarampo ao Brasil, especialmente no ano de 2018^(7)^. O país perdeu o título de área livre de circulação do vírus autóctone que havia recebido em 2016^(5)^.

De acordo com estudo ecológico^(8)^ envolvendo as análises de coberturas vacinais em crianças com até 12 meses de idade no período de 2013 a 2020, é possível notar a redução expressiva deste indicador ao longo dos anos, se intensificando ainda mais com a pandemia de Covid-19. Neste contexto, observa-se que a cobertura vacinal já apresentava valores abaixo das metas estabelecidas pelo Ministério da Saúde e, a cada ano, a queda se acentua^(8)^.

A redução dos índices de coberturas vacinais, nos últimos anos, não pode ser atribuída a uma única causa^(9)^. Diversos fatores podem afetar a queda das coberturas vacinais em crianças. Pode-se destacar os fatores contextuais, históricos, socioculturais, ambientais, do sistema de saúde, econômicos, políticos e até mesmo fatores individuais. Compreender estas associações é um processo complexo^(7,10-12)^, mas torna-se imprescindível entender os múltiplos fatores para desenvolver estratégias visando o aumento da cobertura vacinal.

Dado o complexo cenário de queda da cobertura vacinal que se instala no Brasil e mundialmente (e que foi ainda mais impactado com a pandemia de Covid-19), com motivos descritos para o insucesso das atuais medidas, e com a comprovação das dificuldades enfrentadas pelo PNI, existe uma crescente preocupação com a criação de estratégias que visem o aumento da cobertura vacinal entre as crianças, especialmente com a melhoria do acesso aos serviços de saúde, indiferente do tipo de vacina aplicada e das características sociais das crianças menores^(13)^.

As intervenções para melhorar os resultados da vacinação são comumente agrupadas naquelas que visam a prestação ou oferta de serviços de saúde (por exemplo, melhorar o treinamento de recursos humanos, logística, manutenção da cadeia de frio e armazenamento de vacinas, financiamento eficaz, monitoramento e supervisão de apoio) e aquelas que estimulam a demanda por vacinas (incentivos monetários ou alimentares, transferência de conhecimento ou campanhas de comunicação). Localizou-se revisões sistemáticas a nível internacional que evidenciaram estes estudos^(14,15)^, mas com limitações na qualidade e no desenho^(16)^. A Secretaria de Estado de Saúde do Estado de Goiás, Brasil, divulgou recentemente um *overview* de revisões sistemáticas que abrangem estratégias para aumentar a cobertura vacinal. Dentre as 18 revisões sistemáticas publicadas entre 2018 e 2021, não foram identificadas revisões sobre essa temática no Brasil, o que corrobora a necessidade de estudos que implementaram estratégias a nível nacional^(17)^.

Este trabalho pode, portanto, subsidiar os gestores e profissionais de saúde envolvidos com a vacinação na construção do planejamento e implementação de suas ações, visto que o PNI brasileiro é considerado um dos mais completos do mundo^(7)^.

## OBJETIVOS

Identificar quais foram as estratégias presentes na literatura para ampliação da cobertura vacinal em crianças no Brasil, sem restrição de período. Se justifica principalmente pelo cenário atual de queda de coberturas vacinais.

## MÉTODOS

Trata-se de uma revisão sistemática de literatura, realizada de acordo com as recomendações do *Cochrane Handbook for Systematic Intervention Reviews* ^(18)^ e relatada com base nas etapas recomendadas pelo PRISMA^(19)^. O protocolo do estudo foi registrado na Plataforma OSF^®^ (*Open Science Framework*), sob o identificador 10.17605/OSF.IO/XHM6P: DOI^(20)^.

A questão de pesquisa desta revisão foi: “Quais são as estratégias utilizadas para ampliar as taxas de vacinação em crianças no Brasil?”. Para adaptar a questão de pesquisa à construção da estratégia de busca, foi utilizada a estrutura PCC (População - Conceito - Contexto)^(21)^ (Tabela S1 - Dados da Pesquisa). Levando-se em consideração o acrônimo P (População), foram consideradas as crianças. Para o acrônimo C (Conceito), foram utilizados os descritores de planejamento de saúde, plano de ação e gestão municipal. Para o acrônimo C (Contexto), foram utilizados os descritores de vacinação, imunização, coberturas vacinais, Programa de Imunização e Brasil.

A opção por considerar apenas estratégias implementadas no Brasil se deu em razão de o PNI brasileiro ser considerado um dos mais completos do mundo, com reconhecimento internacional, com elevadas coberturas vacinais para diversos agravos de importância para a saúde pública, assim como pela implementação de estratégias e logística de alcance de imunização para toda a população brasileira que está inserida em um extenso território continental^(7)^.

### Estratégia de Busca

A estratégia de busca foi realizada com os termos da PCC adaptados às diferentes bases de dados Pubmed (MEDLINE), Embase e Scopus (Tabela S2 - Dados da Pesquisa). Os termos da metodologia PCC, quando disponíveis como termos indexadores (MESH e Entree), foram utilizados concomitantemente aos termos texto^(22,23)^. As buscas foram realizadas sem restrições de ano e idioma, no mês de dezembro de 2022, e atualizada em setembro de 2023 por meio de nova busca. Os termos de pesquisa sinônimos incluídos foram separados por operadores boleanos “*OR*” e cada grupo de termos sinônimos da PCC agrupados por “*AND*”.

As chaves de busca utilizadas em cada base de dados estão descritas abaixo:

Pubmed: (((“*Child*”[MeSH *Terms*] *OR* “*Child*”[MeSH *Terms*] *OR* “*Child*”[TW] *OR* “*children*”[TW] *OR* “*child s*”[TW] *OR* “*children s*”[TW] *OR* “*childrens*”[TW] *OR* “*childs*”[TW] *OR* “*Child*”[MeSH *Terms*] *OR* “*Child*”[TW] *OR* “*children*”[TW] *OR* “*child s*”[TW] *OR* “*children s*”[TW] *OR* “*childrens*”[TW] *OR* “*childs*”[TW] *OR* “*Infant*”[MeSH *Terms*] *OR* “*Infant*”[MeSH *Terms*] *OR* “*Infant*”[TW] *OR* “*infants*”[TW] *OR* “*infant s*”[TW] *OR* “*Infant*”[MeSH *Terms*] *OR* “*Infant*”[TW] *OR* “*infants*”[TW] *OR* “*infant s*”[TW] *OR* “*nurseling*”[TW] *OR* “*nursling*”[TW] *OR* “*nurslings*”[TW])) *AND* (((“*Health Planning*” *OR* “*Health Management*” *OR* “*Municipal Management*” *OR* “*Action Plan*”) *AND* (“*Vaccination*”[Mesh] *OR* (*Vaccination*) *OR* “*Immunization*”[Mesh] *OR* (*Immunization*) *OR* (*Vaccination Coverage*) *OR* (*Immunization*) *OR* (*Active Immunization*) *OR* (*Immunization Coverage*) *OR* “*Mass Vaccination*”[Mesh] *OR* (*Mass Vaccination*) *OR* “*Vaccination Coverage*”[Mesh] *OR* (*Vaccination Coverage*))) *OR* ((*Immunization strategies*) *OR* (*Immunization programs*) *OR* “*Immunization Programs*”[Mesh]))) *AND* (“*Brazil*”[Mesh] *OR* (*Brazil*) *OR* (Brasil))

Embase: (‘*child’*/*syn OR* ‘*infant’*/*syn*) *AND* (‘*vaccination’*/*syn OR* ‘*immunization’*/*syn OR* ‘*mass immunization*’/*syn OR* ‘*preventive health service*’/*syn*) *AND* (‘*Brazil*’/*syn*)

Scopus: ( *ALL* ( *children OR child OR infant* ) ) *AND* ( *ALL* ( “*Health Planning*” *OR* “*Health Management*” *OR* “*Municipal Management*” *OR* “*Action Plan*” ) ) *AND* ( *ALL* ( “*Vaccination*” *OR* “*Immunization*” *OR* “*Vaccination Coverage*” *OR* “*Active Immunization*” *OR* “*Immunization Coverage*” *OR* “*Mass Vaccination*” ) ) *AND* (*ALL* (*Brazil*))

### Critérios de Elegibilidade

Foram incluídos estudos observacionais que continham estratégias de aumento de coberturas vacinais em crianças e que foram realizados no Brasil, sem restrição de período. Os critérios de exclusão foram (1) Revisões sistemáticas e metanálises; (2) Ensaios clínicos; (3) Estudos experimentais; (4) Estudos de caso-controle; (5) Cartas ao Editor; (6) Estudos que não apresentaram estratégias de intervenção no aumento de coberturas vacinais de crianças.

### Seleção e extração dos Estudos

Dois revisores independentes (JFAS e TPRS) examinaram os títulos e resumos dos artigos identificados por meio da plataforma Rayyan^®^. Os estudos que atenderam aos critérios de inclusão foram submetidos à leitura do texto completo. As discrepâncias foram resolvidas por consenso. Na persistência da dúvida, um terceiro (CKD) e quarto revisor (FPM) foram acionados. Os trabalhos que atenderam aos critérios de elegibilidade foram incluídos na revisão. Os mesmos dois revisores realizaram a extração de dados por meio da metodologia SWiM.

A diretriz SWiM possui uma lista de verificação de nove itens para promover relatórios transparentes: (1) Agrupamento de estudos para síntese; (2) Descrição da métrica padronizada e os métodos de transformação usados; (3) Descrição dos métodos de síntese; (4) Critérios utilizados para priorizar resultados para resumo e síntese; (5) Investigação da heterogeneidade nos efeitos relatados; (6) Certeza de evidência; (7) Métodos de apresentação dos dados; (8) Relatórios de resultados e (9) Limitações da síntese^(24)^.

### Qualidade metodológica

Todos os estudos incluídos foram avaliados pelos autores (JFAS e TPRS) de forma independente quanto à qualidade metodológica e risco de viés, utilizando a escala de Newcastle-Ottawa, adaptada para estudos observacionais^(21,23)^. Realizou-se uma avaliação dos estudos usando os seguintes domínios: (i) tamanho e representatividade da amostra (0-4 pontos); (ii) comparabilidade entre participantes (0-2 pontos) e (iii) descrição da estratégia de imunização (0-3 pontos). Considerou-se um estudo de baixa qualidade se recebeu menos de sete estrelas e de alta qualidade se recebeu sete estrelas ou mais (Quadro 3).

### Análise dos dados

Os dados foram agrupados por síntese narrativa e os resultados formatados em tabelas de forma descritiva, utilizando a metodologia SWiM.

## RESULTADOS

A estratégia de busca recuperou 4.824 citações, das quais 1.253 foram excluídas por duplicidade. Assim, 3.571 títulos e resumos foram avaliados com o auxílio de ferramenta de automação (Rayyan^®^). Após esta etapa, 48 estudos foram incluídos e procurados para recuperação. Deste total, 7 não foram encontrados, permanecendo 41 estudos para leitura completa. Finalmente, 6 estudos foram incluídos na Revisão Sistemática (Figura 1 e Quadro 1).

**Quadro 1:** Características de autoria, localização, tamanho amostral, população dos estudos, idade e imunobiológicos analisados

Autor, Ano	Localização	Tamanho Amostral	População Estudada	Idade	Imunobiológicos analisados
Andrade *et al*., 2012^(25)^	Brasil	7.550 crianças	(1) famílias classificadas na linha de pobreza, com filhos menores de 15 anos ou gestantes; (2) famílias classificadas na linha de extrema pobreza com ou sem filhos.	Crianças de 0 a 6 anos	Imunobiológicos que compunham o Calendário Básico de Vacinação das Crianças no ano do estudo, até 6 anos de idade.
Barcelos *et al.,* 2021^(26)^	30 municípios dos estados da Bahia, Ceará, Goiás, Pará, Pernambuco e São Paulo	3.242 crianças (T0) e 3.008 crianças (T1)	Crianças e gestantes elegíveis para o Programa Criança Feliz.	T0 - menos de 12 meses de vida; T1 - mediana de 12 meses	Imunobiológicos que compunham o Calendário Básico de Vacinação das Crianças no ano do estudo, até os 15 meses de idade.
Costa *et al.,* 2020^(29)^	Município de São Paulo	151 crianças antes da intervenção e 145 depois da intervenção.	Integrantes do Centro de Educação Infantil de São Paulo (CEI)	Crianças de 0 a 4 anos de idade.	Imunobiológicos que compunham o Calendário Básico de Vacinação das Crianças no ano do estudo, até os 4 anos de idade.
Moura *et al*., 2018^(27)^	Estado do Ceará	52.216 cadernetas de vacinação	População residente no Estado do Ceará	Crianças de 6 meses a menores de 5 anos de idade	Vacinas dupla viral, tríplice viral e tetraviral.
Silva *et al*., 2020^(30)^	São Luís (MA) e Ribeirão Preto (SP)	532 crianças em Ribeirão Preto e 1.229 em São Luís	Beneficiários do Programa Bolsa Família	Crianças de 13 a 35 meses	Imunobiológicos que compunham o Calendário Básico de Vacinação das Crianças no ano do estudo, até o primeiro ano de vida.
Cufino Svitone *et al*., 2000^(28)^	Ceará	-	População do estado do Ceará coberta pelas Equipes de Saúde da Família	-	-

**Figura 1:** Processo de seleção de artigos

*Adaptado de: Page MJ, McKenzie JE, Bossuyt PM, Boutron I, Hoffmann TC, Mulrow CD, et al. The PRISMA 2020 statement: an updated guideline for reporting systematic reviews. BMJ 2021;372:n71. doi: 10.1136/bmj.n7*

Foram analisados os diversos imunobiológicos na faixa-etária das crianças, partindo de 0 até 6 anos de idade. Os estudos levaram em consideração as seguintes localidades: Brasil^(25)^; estado do Ceará^(26-28)^, Bahia^(26)^, Goiás^(26)^, Pará^(26)^, Pernambuco^(26)^, São Paulo^(26,29)^; municípios de São Luís (MA) e Ribeirão Preto (SP)^(30)^ (Quadro 1).

Dos 6 artigos incluídos, 50% abordaram estudos relacionados ao Programa Bolsa Família (PBF) e avaliação das coberturas vacinais^(25,26,30)^. Os outros exploraram estratégias de abordagem direta aos pais^(29)^, Monitoramento Rápido de Vacinação (MRV)^(27)^ e Programa de Agentes Comunitários de Saúde (PACS)^(28)^ (Quadro 2).

**Quadro 2:** Caracterização das estratégias relatadas para ampliação de coberturas vacinais de crianças

Autor, Ano	Estratégia	Ator (quem realizou)	Estratégias relatadas para ampliação da cobertura vacinal	Nível da estratégia	Principais resultados
Andrade *et al*., 2012^(25)^	Programa de Transferência Condicional de Renda (CCT)/Programa Bolsa Família (PBF).	Governo Federal	Estabelecimento de condicionalidades nos programas de transferência de renda de forma que as famílias recebam o pagamento em dinheiro apenas se cumprirem determinados requisitos, como manter o cartão de vacinação das crianças em dia.	Macropolítica	O PBF não influenciou a condição vacinal das crianças em 2005. Efeitos foram encontrados apenas na região Sudeste/Sul. Em média, o efeito do programa representa um aumento de 7% na imunização, com 10% sendo significativo nesta região.
Barcelos *et al.,* 2021^(26)^	Programa de Transferência Condicional de Renda (CCT)/Programa Bolsa Família (PBF) /Programa Criança Feliz (PCF)	Ministério da Cidadania (MCid)	O PCF é direcionado a crianças menores de 3 anos de idade filhos de famílias beneficiadas pelo PBF. Para recebimento do pagamento em dinheiro, os pais cadastrados devem levar todos os filhos menores de 7 anos a unidade de saúde para imunização completa.	Macropolítica	Baixo percentual de crianças com vacinação adequada, tanto no primeiro quanto no segundo ano de vida. O pertencer ao quintil mais rico - no âmbito de uma amostra predominantemente pobre, foi fator associado a maiores proporções de vacinação adequada no T0. Em T1, a cobertura vacinal no grupo PCF foi maior do que no controle.
Costa *et al*., 2020^(29)^	Abordagem direta aos pais	Centro de Educação Infantil de São Paulo (CEI)	Envio de um lembrete às famílias por meio da agenda da criança sobre a situação vacinal e os cuidados que promovem a sua vacinação.	Micropolítica	Houve diferença na prevalência da completude vacinal das crianças em atraso após a intervenção (aumento de 11,6% - passou de 81,5% para 93,1%). Envio de lembrete aos pais: aumento da conclusão da vacinação das crianças.
Moura *et al.,* 2018^(27)^	Monitoramento Rápido de Vacinação (MRV)	Secretaria Estadual de Saúde e municípios do Ceará	O MRV se caracteriza por buscar a cobertura vacinal em visitas domiciliares para verificação do comprovante de vacinação.	Macropolítica	Identificada 1,6% de crianças não vacinadas contra o sarampo. Após o MRV apenas quatro municípios (Baturité, Itapipoca, Brejo Santo e Crato) não atingiram a cobertura vacinal para a primeira dose, e duas CRES (Itapipoca e Russas) não atingiram a cobertura vacinal para a segunda dose.
Silva *et al*., 2020^(30)^	Programa de Transferência Condicional de Renda (CCT)/Programa Bolsa Família (PBF).	Governo Federal	Estabelecimento de condicionalidades nos programas de transferência de renda de forma que as famílias recebam o pagamento em dinheiro apenas se cumprirem determinados requisitos, como manter o cartão de vacinação das crianças em dia	Macropolítica	Ser beneficiário do PBF não teve efeito sobre a vacinação infantil em crianças pertencentes a famílias de baixa renda, tanto em São Luís quanto em Ribeirão Preto.
Cufino Svitone *et al*., 2000^(28)^	Programa de Agentes Comunitários de Saúde (PACS)	Governo do Estado do Ceará	Atuação dos Agentes Comunitários de Saúde (ACS) na realização de visitas domiciliares e ações de prevenção de doenças e promoção à saúde na comunidade.	Macropolítica	Rápido declínio na mortalidade infantil, aumento na imunização, identificação de gargalos que limitam a utilização de outros recursos médicos e intervenções oportunas em tempos de crie. Identificação rápida de áreas com baixas coberturas vacinais e resolução dos problemas.

**Quadro 3:** Escala de Newcastle-Ottawa para análise da qualidade dos estudos

Autor, Ano	Seleção (máximo quatro estrelas)	Comparabilidade (máximo duas estrelas)	Avaliação do Resultado (máximo três estrelas)	Qualidade
Andrade *et al*., 2012^(25)^	^****^	^**^	^***^	Alta
Barcelos *et al*., 2021^(26)^	^****^	^**^	^***^	Alta
Costa *et al*., 2020^(29)^	^**^	^*^	^**^	Baixa
Moura *et al.,* 2018^(27)^	^****^	^*^	^***^	Alta
Silva *et al*., 2020^(30)^	^****^	^**^	^***^	Alta
Cufino Svitone *et al*., 2000^(28)^	^**^	^*^	^***^	Baixa

O PBF, MRV e PACS representam estratégias que são consideradas macropolíticas, ou seja, há uma diretriz institucional com instrumentos e ferramentas padronizados para execução em todo o território do país. A estratégia relacionada à abordagem direta aos pais representou uma micropolítica, ou seja, as decisões foram tomadas por indivíduos, agentes de saúde do seu território, com base em seus contextos e realidades locais^(31)^.

Dentre as estratégias de macropolíticas, destaca-se o PBF que foi abordado em três estudos^(25,26,30)^. Todos avaliaram as condicionalidades do PBF com a completude vacinal das crianças participantes (Quadro 2). Nos três estudos os resultados foram semelhantes em relação à não influência do PBF na melhoria da cobertura vacinal, mesmo sendo uma das condicionalidades do programa. No estudo de Barcelos e colaboradores^(26)^, além de analisar o percentual de crianças com vacinação adequada, realizou-se também uma estratificação socioeconômica que demonstrou que pertencer ao quintil mais rico - no âmbito de uma amostra predominantemente pobre - foi um fator associado a maiores proporções de vacinação adequada. O que se pode afirmar, com base nos três estudos, é que é importante melhorar tanto o acompanhamento das condicionalidades do programa quanto o monitoramento da situação vacinal, uma vez que os percentuais de incompletude vacinal em crianças cujas famílias recebem o PBF foram elevados.

No estudo de Moura e colaboradores^(27)^, o MRV foi definido como uma atividade de supervisão das ações de vacinação recomendada pela Organização Pan-Americana da Saúde (OPAS) desde a década de 1980, adotada em vários países das Américas. O MRV permitiu conhecer os motivos da não vacinação, sendo alguns deles de responsabilidade dos cuidadores das crianças (recusa, perda da caderneta de vacinação, falta de tempo) e outros por responsabilidade da gestão (dificuldade de acesso aos locais de vacinação, falta de agendamento da vacina na Caderneta de Saúde da Criança, ausência de garantia do estoque do imunobiológico, falta de flexibilidade nos horários de funcionamento das unidades de saúde)^(27)^ (Quadro 2).

O estudo de Cufino Svitone e colaboradores^(28)^ relatou a estratégia precursora no Estado do Ceará que deu origem ao Programa de Agentes Comunitários de Saúde (PACS). Como resultados, o PACS foi amplamente empregado e possibilitou a melhoria significativa dos indicadores de saúde materno-infantil entre os anos de 1987 e 1994. O monitoramento populacional possibilitou a identificação rápida de áreas com baixa cobertura vacinal e o enfrentamento do problema. Entre as chaves do sucesso do Programa estão a seleção criteriosa dos agentes e a qualidade de sua supervisão e treinamento, feitas pelos enfermeiros. Tornou-se modelo para oito estados brasileiros vizinhos^(28)^ (Quadro 2).

A estratégia de micropolítica foi relatada por Costa e colaboradores^(29)^, e o objetivo foi avaliar a completude e o atraso vacinal das crianças de um centro de educação infantil (localizado em uma região de alta vulnerabilidade social), antes e após uma intervenção educativa com as famílias. A intervenção consistiu no envio de um lembrete às famílias por meio da agenda da criança sobre a situação vacinal da mesma. Caso fosse verificada a completude da situação vacinal, a família era parabenizada pelos cuidados com a criança e informada quanto às próximas vacinas a serem tomadas. Caso fosse verificado atraso na situação vacinal, a família era informada sobre o fato e orientada a procurar um serviço de saúde para regularização^(29)^ (Quadro 2).

Com relação à avaliação da qualidade metodológica dos estudos, 4 estudos^(25-27,30)^ foram classificados em ≥ 7 de 10 estrelas, obtendo a classificação de qualidade alta. Os estudos de Costa *et al*.^(29)^ e Cufino Svitone *et al*.^(28)^ apresentaram qualidade metodológica baixa, principalmente em relação aos critérios de seleção (representatividade) e comparabilidade (neste caso, para qualquer fator adicional).

## DISCUSSÃO

O número restrito de estudos publicados no Brasil sobre estratégias para aumento de coberturas vacinais em crianças ficou evidenciado por meio desta revisão sistemática. A maior parte dos estudos encontrados tratou de uma estratégia específica, que é o Programa Bolsa Família (PBF)^(25,26,30)^.

O PBF teve início em 2003, sendo um programa de transferência de renda criado pelo Governo Federal Brasileiro, com o objetivo de promover a redução imediata da pobreza por meio de transferências diretas de renda^(31)^. Possui um caráter inovador devido à possibilidade de quebra do ciclo intergeracional da pobreza historicamente experenciado pela população brasileira, em especial a população de cor de pele preta e parda. Para isto foram definidas condicionalidades que reforçam o exercício dos direitos sociais nas áreas de saúde e educação, permitindo, em grande parte, a luta contra a pobreza no futuro. Dentre as condicionalidades está o cumprimento do Calendário Nacional de Vacinação da Criança^(32)^.

Os principais resultados dos três estudos indicaram que o PBF não contribuiu para a melhora da situação vacinal das crianças, apesar de o cumprimento do Calendário Vacinal definido pelo Ministério da Saúde ser uma das condicionalidades para recebimento do benefício^(25,26,30)^.

A defesa da exigência de condicionalidades para o PBF tem sido sustentada pelo argumento de que ela propicia acesso às políticas de saúde, educação e assistência social àquelas famílias beneficiárias destes programas. No entanto, o que se observa é que as políticas públicas são insuficientes para a população, ou seja, não é porque esses beneficiários têm que cumprir determinadas ações que as políticas estarão plenamente prontas para receber esta população, como deveria ser^(33)^ . Tem-se discutido que as condicionantes definidas para as políticas públicas facilitam o acesso das camadas mais abastadas da sociedade aos serviços básicos de saúde e educação, entretanto interroga-se sobre a capacidade destes serviços de absorverem (e fiscalizarem) adequadamente o aumento da demanda resultante do cumprimento das condicionalidades^(34)^.

Os resultados encontrados sobre o cumprimento das condicionalidades do PBF no Brasil diferem muito dos que foram observados no México, onde são sistematicamente verificadas. Por exemplo, o Programa Mexicano exige que crianças com idade entre 24 e 60 meses compareçam obrigatoriamente às clínicas de monitoramento nutricional a cada quatro meses, além do envolvimento em atividades preventivas de saúde e nutrição^(35,36)^. Os autores reforçaram a necessidade de investigar os motivos do descumprimento das condicionalidades do Programa e os custos de fiscalização associados a tais estratégias de políticas públicas^(25)^.

No estudo de Silva e colaboradores^(30)^, tanto em São Luís, Brasil, como em Ribeirão Preto, Brasil, entre as crianças pertencentes a famílias de baixa renda, ser beneficiário do PBF também não teve efeito no esquema vacinal infantil. Este estudo também citou o Programa Mexicano como modelo que tem demonstrado melhoria de resultados de saúde, crescimento e desenvolvimento infantil.

Barcelos e colaboradores^(26)^ considerou algumas hipóteses para a diferença encontrada nas prevalências de cobertura vacinal do seu estudo: parte da diminuição na cobertura seria reflexo da falta de vacinas no período (especialmente a pentavalente); há características locais na implementação do Programa de Imunizações entre estados e municípios (oferta de vacinas, acesso aos serviços de saúde e regularidade do registro na Caderneta de Saúde da Criança), bem como na adesão das famílias à vacinação de suas crianças. Considerando a vacinação uma das condicionalidades do PBF, as diferenças também podem ser atribuídas a características locais de implementação do Programa. Os achados do estudo sugerem a necessidade de uma avaliação detalhada da adesão das famílias à condicionalidade de manter o esquema de vacinação de suas crianças atualizado^(26)^.

Um estudo que pesquisou o surgimento da hesitação vacinal entre os brasileiros de classe alta concluiu que, nos últimos anos, o declínio mais rápido da cobertura vacinal está acontecendo entre crianças de famílias ricas, provavelmente relacionado à hesitação vacinal (ou seja, um “atraso na aceitação ou recusa da vacinação apesar da disponibilidade de serviços de vacinação”)^(37)^. O crescimento das mídias sociais e a disseminação de notícias falsas, aliada à atual crise de financiamento dos serviços de saúde, representa um grande desafio para o aumento das coberturas vacinais^(38)^.

Apesar de estarem entre as intervenções políticas com maior impacto estimado na queda da mortalidade infantil no país, o PBF e a Estratégia Saúde da Família (ESF) são afetados diretamente pela austeridade fiscal, que reduzirão substancialmente os gastos com programas de assistência social como percentual do PIB do país^(39)^. A Emenda Constitucional 95 (EC 95) é a medida de austeridade mais impactante, que não se limita à crise econômica e ainda perdurará por 20 anos, causando impacto importante no já frágil estado de bem-estar social no Brasil^(40)^. A redução do investimento na saúde e nos programas de assistência social (como o PBF) agravou a desigualdade social no país, aumentou a concentração de renda e deteriorou os indicadores de saúde, entre eles, o índice de cobertura vacinal^(40,41)^.

No estudo de Moura e colaboradores^(27)^, o Monitoramento Rápido de Vacinação (MRV) é definido como uma atividade de supervisão das ações de vacinação recomendada pela Organização Pan-Americana da Saúde (OPAS) desde a década de 1980, adotada em vários países das Américas. Consiste em buscar a cobertura vacinal em visitas domiciliares para verificação do comprovante de vacinação. O número de domicílios visitados baseia-se no tamanho da população-alvo e no número de salas de vacina em cada município. Os dados foram analisados por meio de um MRV implementado após uma campanha de vacinação indiscriminada contra sarampo no estado do Ceará, em novembro de 2014, obtendo resultados satisfatórios.

Outro estudo descritivo, também utilizando dados do Monitoramento Rápido de Cobertura (MRC) após campanhas de vacinação para eliminação do sarampo e da rubéola nos anos de 2008, 2011 e 2012 no Brasil (crianças, adolescentes e adultos), reiterou a importância de manter esta estratégia para avaliação das coberturas vacinais, principalmente após intensificação das ações de vacinação. O MRC é capaz de resgatar não vacinados e direcionar as intervenções, sobretudo no âmbito local. Fornece, em curto espaço de tempo, a situação vacinal da população-alvo entrevistada, e a baixo custo, quando comparado aos custos dos inquéritos de cobertura e do censo vacinal^(3)^.

A estratégia de busca ativa de faltosos é citada como uma das mais relevantes para gerar impacto positivo no aumento de coberturas vacinais. É uma atribuição dos profissionais da equipe de saúde da família e tem grande relevância no monitoramento dos usuários em situações de risco e no acompanhamento da população em relação ao Calendário Vacinal^(42)^.

O estudo de Cufino Svitone e colaboradores^(28)^ traz um relato das estratégias utilizadas para implementação do Programa de Agentes Comunitários de Saúde (PACS) no Estado do Ceará, ressaltando a melhoria das coberturas vacinais após a utilização desta estratégia. Ao longo do processo de construção do SUS, é inegável a importância da implantação do PACS na construção do elo entre a comunidade e os serviços de saúde^(43)^. O ACS possui como atributos do seu trabalho a competência cultural, a orientação comunitária e a construção do vínculo, relacionando-se cotidianamente com as famílias do seu território e compartilhando saberes técnicos e populares^(44,45)^.

Um estudo^(46)^ mensurou o conhecimento dos ACS sobre o calendário vacinal infantil de crianças até cinco anos. Foi realizada uma pesquisa quantitativa, descritiva, do tipo antes e depois, com amostra de 25 ACS de duas Unidades Básicas de Saúde de Ananindeua-Pará. Antes da intervenção de capacitação, 76% dos ACS afirmaram que verificam o cartão de vacina da criança e dão orientações às mães; 96% acham que verificar o cartão de vacina da criança faz parte do seu trabalho e todos os ACS (100%) declararam que alguma mãe ou pai já perguntou ao agente sobre o cartão de vacina do seu filho. Estes dados reforçam a importância do papel do ACS na busca ativa e orientação correta sobre a vacinação infantil, portanto, há a necessidade de investimento em qualificação e educação permanente destes profissionais^(46)^.

O estudo de Costa e colaboradores^(29)^ se diferenciou dos demais estudos desta revisão sistemática por abordar uma estratégia de micropolítica, realizada a nível local. O envio de lembretes e folhetos educativos para as famílias permitiu o aumento da completude vacinal do calendário das crianças, corroborando estudos anteriores^(15,28,47,48)^ que implementaram intervenções com o objetivo de compartilhar conhecimento sobre a importância da vacinação e auxiliar os pais a recordarem as datas para vacinar seus filhos. Os achados sugerem que uma intervenção simples e de baixo custo pode ser implementada por enfermeiros que invistam em ações de caráter intersetorial, preconizadas pelo Programa Saúde na Escola^(28)^. A prática da enfermagem na Atenção Primária à Saúde envolve o acompanhamento da situação vacinal em escolas de educação infantil. Trata-se de uma importante estratégia de prevenção de agravos imunopreveníveis e promoção da saúde da criança. Uma revisão sistemática sobre programas de vacinação em escolas em países de alta renda mostrou o protagonismo da enfermagem no acompanhamento da situação vacinal, bem como na aplicação dos imunobiológicos e na comunicação com os pais^(49)^.

### Limitações do estudo

As limitações deste estudo consistem em ter diferentes tamanhos de amostra e o fato de serem realizadas em locais diferentes (o que dificulta a comparação entre os estudos), mas do mesmo país. Apesar dessas limitações, este estudo apresenta vários pontos fortes, como o uso de uma metodologia rigorosa e a apresentação de populações socioeconomicamente semelhantes. Outra limitação diz respeito à não utilização de outras bases de dados, no entanto, as bases mais reconhecidas na área da saúde foram utilizadas (Pubmed, Embase e Scopus). Novamente ressalta-se que a opção por considerar apenas estratégias implementadas no Brasil se deu em razão de o PNI brasileiro ser considerado um dos mais completos do mundo, com reconhecimento internacional.

### Contribuições para a Área

Esta revisão sistemática poderá subsidiar profissionais enfermeiros envolvidos com a imunização para a implementação de estratégias de aumento de coberturas vacinais em crianças, diante de um cenário preocupante de retorno de doenças preveníveis por vacina e da escassez de estudos deste tipo na área.

## CONCLUSÕES

Este estudo corroborou a necessidade de publicação de estratégias de micro e macropolíticas para aumento de coberturas vacinais em crianças, especialmente porque é algo inerente dos serviços federais, estaduais e municipais e que necessitam de maior visibilidade nos meios acadêmicos.

O PBF é uma estratégia importante, no entanto, necessita de maior fiscalização e acompanhamento das suas condicionalidades, principalmente do cumprimento do Calendário Vacinal Infantil. As medidas de austeridade fiscal implementadas nos últimos anos impactaram diretamente os bons indicadores deste Programa.

O Monitoramento Rápido de Vacinação ou de Cobertura (MRV/MRC) precisa ser implementado em todas as ações de intensificação vacinal, como estratégia para verificar a cobertura vacinal do território, resgatar não vacinados e entender os motivos de recusa. Um dos pontos essenciais desta estratégia é a realização de busca ativa de faltosos, que também precisa fazer parte da rotina dos serviços de Atenção Primária à Saúde.

A atuação dos ACS é indiscutivelmente uma estratégia essencial para realização de busca ativa de não vacinados e orientação da população, uma vez que representam o elo mais próximo entre o serviço de saúde e a comunidade.

Além das estratégias de macropolíticas relatadas, estratégias de micropolíticas implementadas de acordo com a realidade de cada território também são importantes, a exemplo das ações integradas dos serviços de saúde e a comunidade escolar.

O Estado, enquanto promotor da saúde coletiva, deve prover estratégias de macropolíticas de aumento de coberturas vacinais, garantindo o monitoramento e avaliação constante de sua eficácia e efetividade. Os profissionais de saúde envolvidos com a imunização a nível local, especialmente os enfermeiros, possuem também o dever de promover estratégias baseadas em seus conhecimentos de território, concretizando micropolíticas que poderão ser diferenciais para a mudança do cenário local.

## DISPONIBILIDADE DE DADOS E MATERIAL

https://doi.org/10.48331/scielodata.YRNMB1
